# Distribution of ticks, tick-borne pathogens and the associated local environmental factors including small mammals and livestock, in two French agricultural sites: the OSCAR database

**DOI:** 10.3897/BDJ.8.e50123

**Published:** 2020-05-05

**Authors:** Isabelle Lebert, Albert Agoulon, Suzanne Bastian, Alain Butet, Bruno Cargnelutti, Nicolas Cèbe, Amélie Chastagner, Elsa Léger, Bruno Lourtet, Sébastien Masseglia, Karen D. McCoy, Joël Merlet, Valérie Noël, Grégoire Perez, Denis Picot, Angélique Pion, Valérie Poux, Jean-Luc Rames, Yann Rantier, Hélène Verheyden, Gwenael Vourc'h, Olivier Plantard

**Affiliations:** 1 Université Clermont Auvergne, INRAE, VetAgro Sup, UMR EPIA, F-63122, Saint-Genès Champanelle, France Université Clermont Auvergne, INRAE, VetAgro Sup, UMR EPIA F-63122, Saint-Genès Champanelle France; 2 INRAE, BIOEPAR, Oniris, F-44307, Nantes, France INRAE, BIOEPAR, Oniris F-44307, Nantes France; 3 Université Rennes, CNRS, ECOBIO (Ecosystèmes, biodiversité, évolution) - UMR 6553, 35000 Rennes, France Université Rennes, CNRS, ECOBIO (Ecosystèmes, biodiversité, évolution) - UMR 6553 35000 Rennes France; 4 CEFS, Université de Toulouse, INRAE, F-31326, Castanet-Tolosan, France CEFS, Université de Toulouse, INRAE F-31326, Castanet-Tolosan France; 5 MIVEGEC, Université Montpellier-CNRS-IRD, 911 Avenue Agropolis, 34394 Montpellier, France MIVEGEC, Université Montpellier-CNRS-IRD, 911 Avenue Agropolis 34394 Montpellier France

**Keywords:** Ticks, *Ixodes ricinus*, small mammals, *Apodemus sylvaticus*, *Myodes glareolus*, prevalence, *
Anaplasma
*, *
Borrelia
*, *
Babesia
*, France, forest, agricultural landscapes, livestock, zoonotic disease

## Abstract

**Background:**

In Europe, ticks are major vectors of both human and livestock pathogens (e.g. Lyme disease, granulocytic anaplasmosis, bovine babesiosis). Agricultural landscapes, where animal breeding is a major activity, constitute a mosaic of habitat types of various quality for tick survival and are used at different frequencies by wild and domestic hosts across seasons. This habitat heterogeneity, in time and space, conditions the dynamics of these host-vector-pathogen systems and thus drives acarological risk (defined as the density of infected ticks). The principal objective of the OSCAR project (2011-2016) was to examine the links between this heterogeneity and acarological risk for humans and their domestic animals. Here, we present the data associated with this project.

**New information:**

This paper reports a database on the distribution and densities of *I. ricinus* ticks - the most common tick species in French agricultural landscapes - and the prevalence of three tick-borne pathogens (*Anaplasma phagocytophilum*, *Borrelia* spp. and *Babesia* spp.) in two sites in north-western (“Zone Atelier Armorique”: ZA site) and south-western (“Vallées et Coteaux de Gascogne”: VG site) France. The distribution and density of ticks along a gradient of wooded habitats, as well as biotic variables, such as the presence and abundance of their principal domestic (livestock) and wild hosts (small mammals), were measured from forest cores and edges to more or less isolated hedges, all bordering meadows. Ticks, small mammals and information on local environmental conditions were collected along 90 transects in each of the two sites in spring and autumn 2012 and 2013 and in spring 2014, corresponding to the main periods of tick activity. Local environmental conditions were recorded along each tick and small mammal transect: habitat type, vegetation type and characteristics, slope and traces of livestock presence. Samples consisted of questing ticks collected on the vegetation (mainly *I. ricinus* nymphs), biopsies of captured small mammals and ticks fixed on small mammals. In the VG site, livestock occurrence and abundance were recorded each week along each tick transect.

A total of 29004 questing ticks and 1230 small mammals were captured during the study across the two sites and over the five field campaigns. All questing nymphs (N = 12287) and questing adults (N = 646) were identified to species. Ticks from small mammals (N = 1359) were also identified to life stage. Questing nymphs (N = 4518 *I. ricinus*) and trapped small mammals (N = 908) were analysed for three pathogenic agents: *A. phagocytophilum*, *Borrelia* spp. and *Babesia* spp.

In the VG site, the average prevalence in *I. ricinus* nymphs for *A. phagocytophilum*, *Borrelia* spp. and *Babesia* spp. were, respectively 1.9% [95% CI: 1.2-2.5], 2.5% [95% CI: 1.8-3.2] and 2.7% [95% CI: 2.0-3.4]. In small mammals, no *A. phagocytophilum* was detected, but the prevalence for *Borrelia* spp. was 4.2% [95% CI: 0.9-7.5]. On this site, there was no screening of small mammals for *Babesia* spp. In ZA site, the average prevalence in nymphs for *A. phagocytophilum*, *Borrelia* spp. and *Babesia* were, respectively 2.2% [95% CI: 1.6-2.7], 3.0% [95% CI: 2.3-3.6] and 3.1% [95% CI: 2.5-3.8]. In small mammals, the prevalence of *A. phagocytophilum* and *Borrelia* spp. were, respectively 6.9% [95% CI: 4.9-8.9] and 4.1% [95% CI: 2.7-5.9]. A single animal was found positive for *Babesia microti* at this site amongst the 597 tested.

## Introduction

In agricultural landscapes, where livestock production occupies a large proportion of the surface area, pastures often adjoin different semi-natural ecosystems (forests, woods, hedges). This type of landscape mosaic implies that areas exploited by livestock are also frequently used by a diverse range of wild fauna. Many parasites and pathogens are shared amongst these animal species, even in the absence of direct contact and some may be transmitted between agricultural and semi-natural systems via common arthropod vectors. In France, ticks are major vectors for both human (e.g. *Borrelia burgdorferi* s.l., the agent of Lyme disease) and livestock pathogens (e.g. *Anaplasma phagocytophilum*, inducing granulocytic anaplasmosis or *Babesia divergens*, causing bovine babesiosis), with *Ixodes ricinus* being the most commonly-involved vector.

*I. ricinus* is a three-stage tick that feeds on a wide variety of vertebrate hosts (Sonenshine and Roe 2014, *Bonnet et al. 2016*). While larvae and nymphs may feed on a range of different-sized hosts, adult ticks require a bloodmeal from a larger host, like roe deer *Capreolus capreolus* or domestic animals (Ruiz-Fons et al. 2012). Host species are differently exploited by ticks and display variable susceptibilities to infection by different tick-borne infectious agents, exhibiting different levels of reservoir competence (Ostfeld et al. 2014). The abundance and diversity of different hosts thus influence the density of infected ticks (i.e. the “acarological risk”) and hence the probability of contact with humans and livestock (LoGiudice et al. 2003, Boyard et al. 2007, Takumi et al. 2019).

Agricultural landscapes constitute a mosaic of habitat types that vary in quality for tick survival and host use. The habitat composition of a given plot and its connection with other habitats will determine its use by wild vertebrates and will thus shape local tick-host interactions (Estrada-Peña 2002, Li et al. 2012, Werden et al. 2014, Heylen et al. 2019). Breeding practices and particularly, the management of animal grazing in different types of pastures, will also influence exposure risk of livestock to ticks and the pathogens they carry (Richter and Matuschka 2006, Boyard et al. 2007, Gassner et al. 2008, Agoulon et al. 2012, Ruiz-Fons et al. 2012). However, agricultural mosaics are not temporally fixed and can vary both seasonally and yearly. We are also currently witnessing rapid landscape modifications due to the influence of global changes and particularly those associated with land-use (i.e. relative proportions of breeding/crop surfaces, forest or hedge fragmentation) and climate change (i.e. tick population dynamics are tightly linked to temperature and humidity regimes) (Medlock et al. 2013, Agoulon et al. 2016).

**Figure 1.**
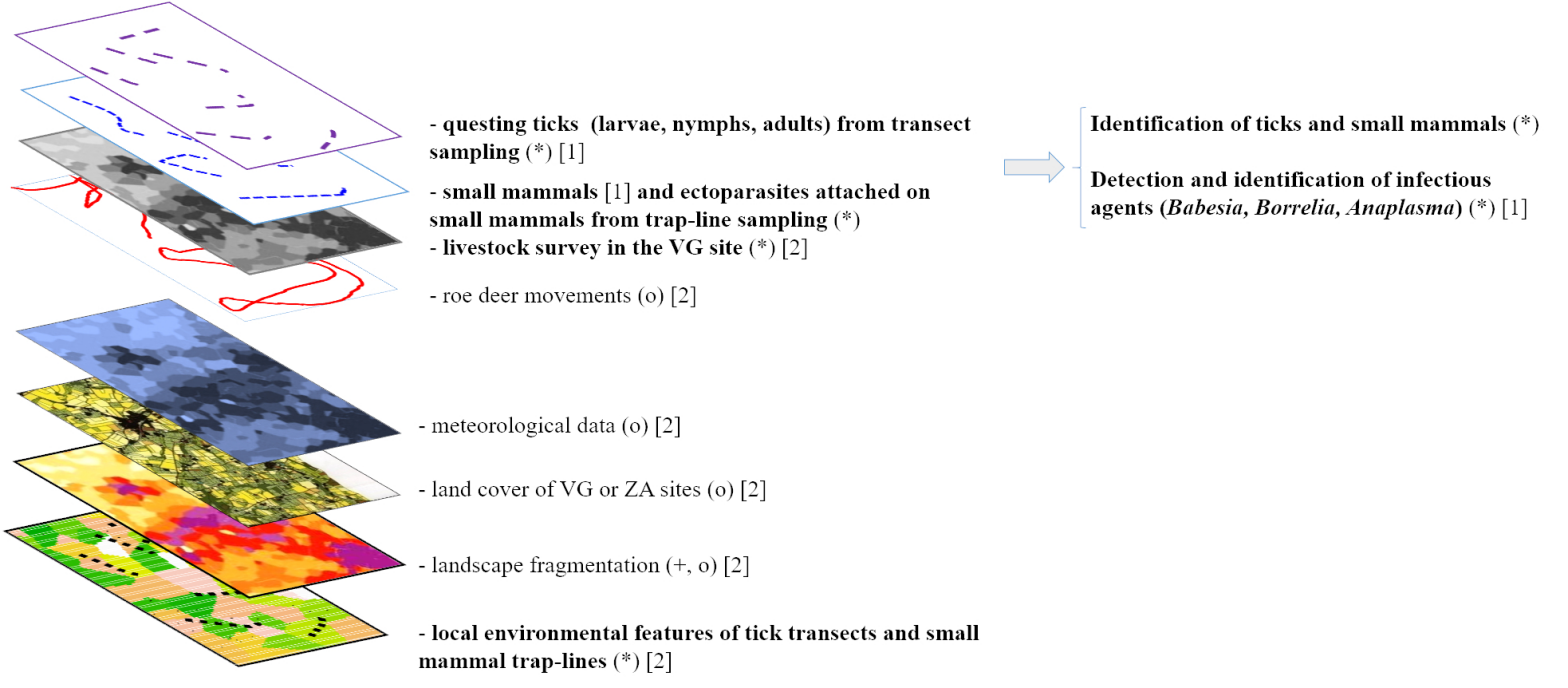
Type of collected data used to study the relationships between landscape structure and acarological risk (i.e. density of infected ticks). Dataset origins: in bold, datasets presented in the datapaper; (*) collected in the field or analysed in the laboratory; (+) calculated from field data; (o) obtained from independent databases. Data uses: [1] response variables: pathogen prevalence in ticks, tick densities, tick population structure; [2] explanatory variables.

The main goal of the OSCAR project (Outil de Simulation Cartographique à l’échelle du paysage Agricole du Risque acarologique / Simulation Tool for Mapping Acarological Risk in Agricultural Landscapes) was to explore the relationships between landscape structure and acarological risk. The study was carried out in two agricultural sites that are part of the International Long-Term Ecological Research (ILTER) network (Zones Ateliers network in France http://www.za-inee.org/en/node/804) and encompass the intrinsic diversity of agricultural landscape features: one LTER site - the “Zone Atelier Armorique” (“ZA site” hereafter) - in north-western France and the second in south-western France in the region of “Vallées et Coteaux de Gascogne” (“VG site” hereafter, belonging to the recently labelled “Zone Atelier PyGar"). Before conducting analyses, the initial task of the OSCAR project consisted of mapping the distribution of ticks, pathogens and the principal domestic (cattle) and wild (small mammals and roe deer) hosts, along a gradient of landscape fragmentation, from forest cores and edges to more or less isolated hedges, all bordering meadows. This paper describes the collected datasets (Fig. 1) (1) on questing tick and small mammal densities, (2) on local environmental conditions (habitat, vegetation and livestock densities) of sampled transects and (3) on pathogen prevalence in ticks and small mammals. Due to time and manpower constraints, we restricted our assessment of tick host species to small mammals, livestock and roe deer, the principal reservoir hosts implicated in disease for production animals. Additional datasets used in some analyses, such as roe deer presence, were not collected in the framework of this study (Fig. 1), but are available elsewhere as outlined in the text.

## Project description

### Personnel

**Laboratories involved**: § BIOEPAR, # CEFS, ¶ MIVEGEC, ‡ EPIA, | ECOBIO, 1 UMR CBGP Montpellier

**Coordinator of the project**: Plantard O. §

**Task managers of the project**: Vourc’h G. ‡ (Sampling, biological analyses and database constitution), McCoy K.D. ¶ (Empirical estimation of factors influencing acarological risk from field data), Hoch T. § (Simulating acarological risk maps according to environmental changes)

**Site managers and contacts for samplings**: Verheyden H. # for the VG (‘‘Vallées et Coteaux de Gascogne’’) LTER site and Butet A. | for the ZA (“Zone Atelier Armorique”) LTER site.

**Data management and Geographic Information System (GIS)**: Agoulon A. §, Bastian S. §, Dorr N. ‡, Lebert I. ‡, Lourtet B. #, Mahé H. §, Rantier Y. |

**Sample collection**

VG site: Angibault J. #, Bailly X. ‡, Bard E. ‡, Bastian S. §, Cargnelutti B. #, Cebe N. #, Chastagner A. ‡, Delrue B. §, Lebert I. ‡, Léger E. ¶, Lourtet B. #, Mahé H. §, Masseglia S. ‡, McCoy K.D. ¶, Merlet J. #, Noël V. ¶, Perez G. §,|, Picot D. #, Pion A. ‡, Poux V. ‡, Quillery E. §, Toty C. ¶, Vaumourin E. ‡, Verheyden H. #, Vincent S. ‡, Vourc'h G. ‡

ZA site: Agoulon A. §, Al Hassan D. |, Armand F. §, Audiart J.-Y. §, Bastian S. §, Billon D. §, Bouju-Albert A. §, Boullot F. §,|, Bruneau A. §, Butet A. |, Daniel J. §, de la Cotte N. §, Delrue B. §, Faille F. §, Gonnet M. ‡, Hermouet A. §, Hoch T. §, Jambon O. |, Jouglin M. §, Lemine-Brahim M. §, Mahé H. §, Moreau E. §, Navarro N. §, Pavel I. §, Perez G. §,|, Plantard O. §, Quillery E. §, Rantier Y. |, Renaud J. §, Roy P. §

**Identification of small mammals**

VG site: Bastian S. §, Butet A. |, Cèbe N. #, Chastagner A. ‡, Cosson J. 1, Léger E. ¶, Masseglia S. ‡, McCoy K.D. ¶, Noël V. ¶, Perez G. §,|, Vaumourin E. ‡, Vourc'h G. ‡

ZA site: Butet A. |, Perez G. §,|, Agoulon A. §, Bastian S. §, Bouju-Albert A. §, Gonnet M. ‡, Hermouet A. §, Moreau E. §, Pavel I. §, Plantard O. §

**Tick identification**

VG site: Pion A. ‡, Poux V. ‡

ZA site: Agoulon A. §, Bouju-Albert A. §, Hermouet A. §, Plantard O. §

**Laboratory analysis**

VG site: Chastagner A. ‡, Masseglia S. ‡, McCoy K.D. ¶, Noël V. ¶, Léger E. ¶

ZA site: Bouju-Albert A. §, Daniel J. §, Faille F. §, Hermouet A. §, Jouglin M. §, Léger E. ¶, McCoy K.D. ¶, Noël V. ¶, Perez G. §,|, Quillery E. §

**Livestock survey**: (VG site only): Angibault J. #, Cargnelutti B. #, Lourtet B. #, Sevila J. #, Verheyden H. #

### Study area description

**LTER site “Vallées et Coteaux de Gascogne” (VG site)**

**Figure 2.**
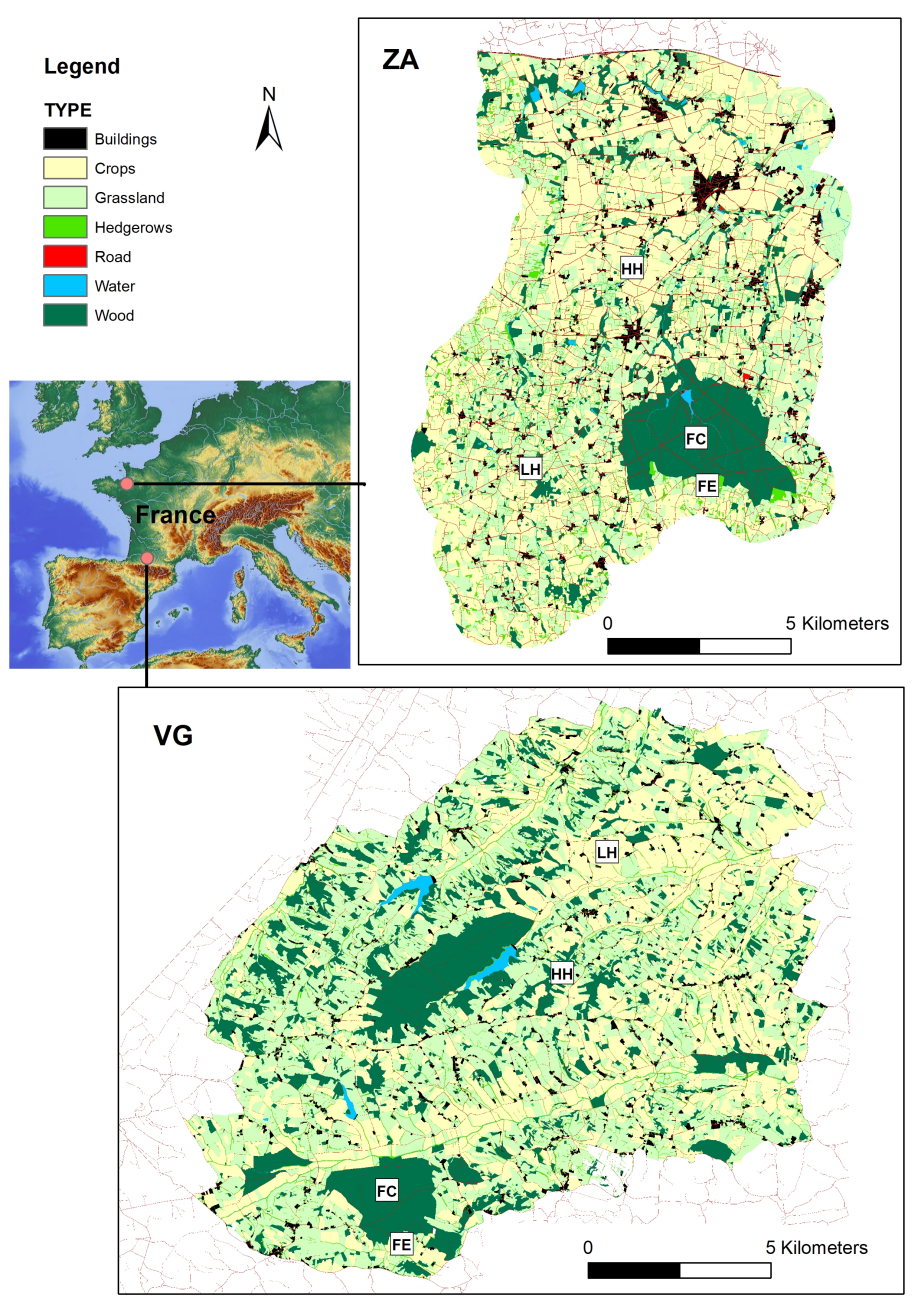
Map of the two studied sites in France: the “Vallées et Coteaux de Gascogne” LTER site (VG) and the “Zone Atelier Armorique” LTER site (ZA). Landscape types: LH, Agricultural landscapes with a Low Hedgerow network density; HH, Agricultural landscapes with a High Hedgerow network density; FE, Forest Edge; FC, Forest Core. A single label per landscape type was drawn on the map (LH, HH, FE, FC), but corresponds to several sampling points in the field. For example, for the FE label, 20 sampling points were designated around the forest (see Fig. 3 for the number of points).

The VG site is a Long Term Ecological Research (LTER) site (referenced as zone atelier Pyrénées Garonne - PYGAR since 2016, https://pygar.omp.eu/), located 75 km from Toulouse in south-western France (43°16'2.64"N, 0°51'51.00"E) (Fig. 2). The area is hilly (altitude 200–400 m above sea level) and dissected by north-south valleys with a mild oceanic climate and summer droughts. Woodland covers 24% of the area with two main forest patches of about 500 and 700 ha, many woods smaller than 50 ha and hedges dominated by *Quercus* spp. Areas dedicated to cultivated crops cover 32% of the main study site. Meadows cover another 40%, amongst which half are grazed by domestic animals (mostly cattle, horses, sheep, but sometimes goats and pigs), either individually or in mixed groups. The roe deer density has been estimated at around 6 roe deer/km^2^ in open areas and more than 30 roe deer/km^2^ in one of the forest areas (Hewison et al. 2007).

**LTER site “Zone atelier Armorique” (ZA site)**

The ZA site (https://osur.univ-rennes1.fr/za-armorique) (Fig. 2) is a labelled LTER area of the CNRS (Centre National de la Recherche Scientifique), where ecological studies have been conducted for over 25 years. It is an agricultural landscape situated in the vicinity of Rennes, which is south of the Mont-Saint-Michel’s Bay (north-east Brittany, Western France) (48°29'22.40"N, 1°33'41.48"W). The area includes a wide array of agricultural landscape features, a forest of about 1000 ha and many woods smaller than 50 ha. The southern part of the site is a fine-grain heterogeneous landscape with a complex network of hedgerows (160 m/ha) enclosing small fields. At the northern part of the site, agricultural intensification has led to a more homogeneous coarse-grain landscape with fewer hedgerows per hectare (70 m/ha) enclosing larger fields. The proportion of grassland is greater in the southern part, whereas fields of maize and cereal dominate the northern part. Small woods are disseminated within both northern and southern areas of the site (Hassan et al. 2012).

## Sampling methods

### Study extent

**Figure 3.**
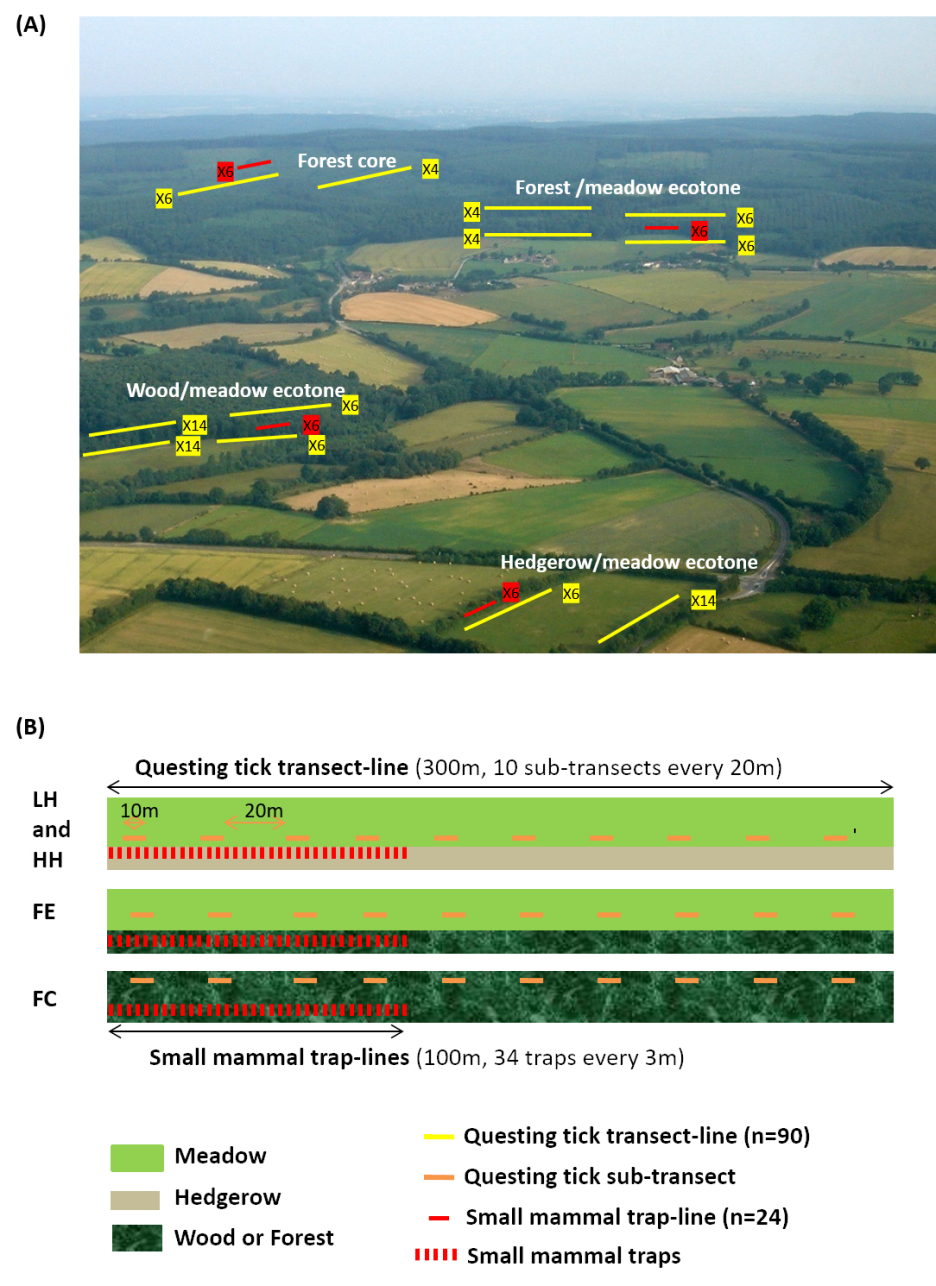
**A** Schematic representation of single and associated sampling transects of ticks and small mammals in the different landscape types. **B** Details of: - questing tick transect-lines, where the drag transect was subdivided into sub-transects - small mammal trap-lines, which contained 34 traps spaced 3 m apart across the initial part of a subset of tick transects Landscape types: LH, Agricultural landscapes with a Low Hedgerow network density HH, Agricultural landscapes with a High Hedgerow network density FE, Forest Edge FC, Forest Core

The study was performed in the two LTER sites (ZA and VG) from 2012 to 2014. Questing ticks and small mammals were sampled during five field campaigns: spring and autumn 2012, spring and autumn 2013 and spring 2014. The sampling design is presented in Fig. 3.

The sampling zones (n = 60) were located in 4 landscape types: Agricultural landscapes with a Low Hedgerow network density (LH); Agricultural landscapes with a High Hedgerow network density (HH); Forest Edge (FE); and Forest Core (FC) (Fig. 3). Small mammals were sampled in 24 zones (amongst the 60 sampling zones), trap-lines being systematically paired with one or two questing tick transect-lines (Fig. 3). Small mammal trap-lines were distributed amongst the four landscape types as follows: six in LH, six in HH, six in FE and six in FC. For each trap-line, 34 traps were spaced 3 m apart along the 100 m line.

Questing ticks were sampled in all 60 zones (including the 24 zones for small mammal sampling). In each zone, one or two transect-lines were defined: 1) a single transect-line was sampled when found along hedgerows and in FC; 2) two transect-lines were run when situated at wood and forest edges (i.e. on either side of the ecotone: one in the meadow and one in the forest) (Fig. 3). This resulted in a total of 90 questing tick transect-lines which were distributed as follows: 30 in LH, 30 in HH, 20 in FE and 10 in FC. For each transect-line, ticks were collected along lines of 300 m, divided into 10 sub-transects of 10 m^2^ each (10 m length x 1 m width), with a space of 20 m between sub-transects (Fig. 3).

The design was fully applied (60 sampling zones) in four campaigns (spring and autumn 2012, spring 2013 and 2014), but only 36 transect lines from the 24 zones used to quantify small mammal presence were sampled during autumn 2013, corresponding to an optimisation of the sampling effort during a less favourable period of tick activity.

### Sampling description

***Recording local environmental conditions***

Georeferencing of sampling locations of ticks (Table 1) and small mammals (Table 2) was obtained in the field using a Trimble GNSS GeoExplorer XT 6000 receiver. A differential correction in post-processing made it possible to obtain decimetric precision. The points obtained were exported in a shape (shp) format and inserted into Geographic Information System (ArcGIS) software. Drawings of the sampling lines were performed on maps by the operators during sampling and were corrected with the GIS database with the help of orthophotos (BD ORTHO®, resolution 50 cm x 50 cm, IGN). During sampling, local environmental conditions were recorded for the questing tick transect-lines, the tick sub-transects and the small mammal trap-lines. The following variables were recorded in the field during tick sampling (Fig. 4) and small mammal sampling (Fig. 5): date and time of the day, habitat type, vegetation type and characteristics, slope, traces of use by livestock. In the VG site, livestock occurrence and abundance were also recorded each week along each tick transect. The livestock survey was only performed in the VG site in association with other research projects and these data were not collected in the ZA site. The data were entered into specific tables of the database (Tables 3, 4, 5, 6).

***Sampling of questing ticks***

**Figure 6.**
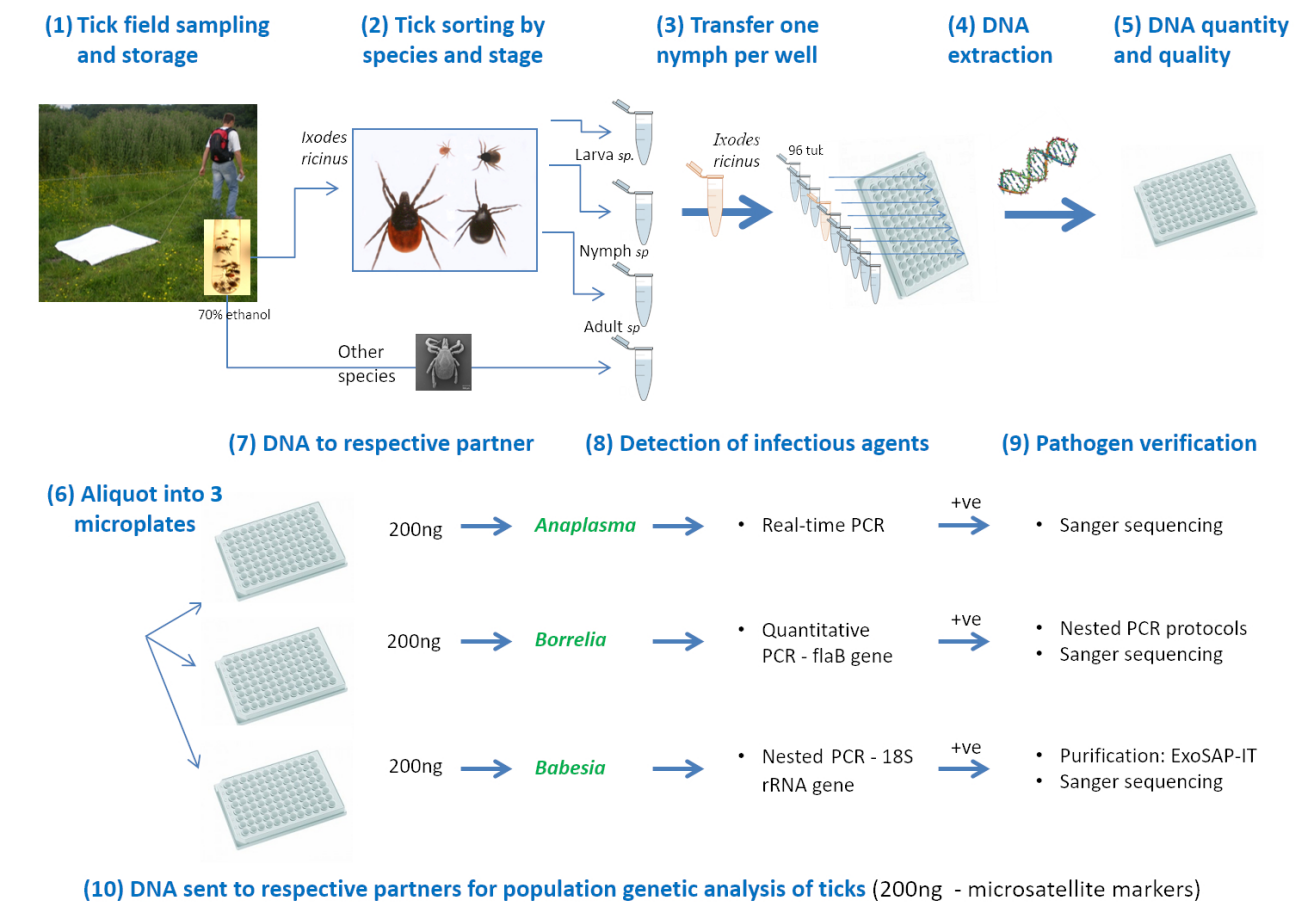
Molecular analyses of ticks; +ve, positive sample.

Questing ticks (Fig. 3) were sampled by flagging (Boyard et al. 2007). In each sub-transect, a 1x1 m white flannel cloth (or ‘flag’) was slowly dragged (0.5 m/s) along 9 m (explored surface of 10 m^2^) across the lower vegetation and leaf-litter (Agoulon et al. 2012). Ticks were counted, collected from the flag and stored in 70% ethanol for later identification (life stage and species) and detection of infectious agents using molecular analyses (Fig. 6, Table 7). Tick identifications were performed using a binocular microscope, according to Pérez-Eid (2007).

***Sampling of small mammals***

**Figure 7.**
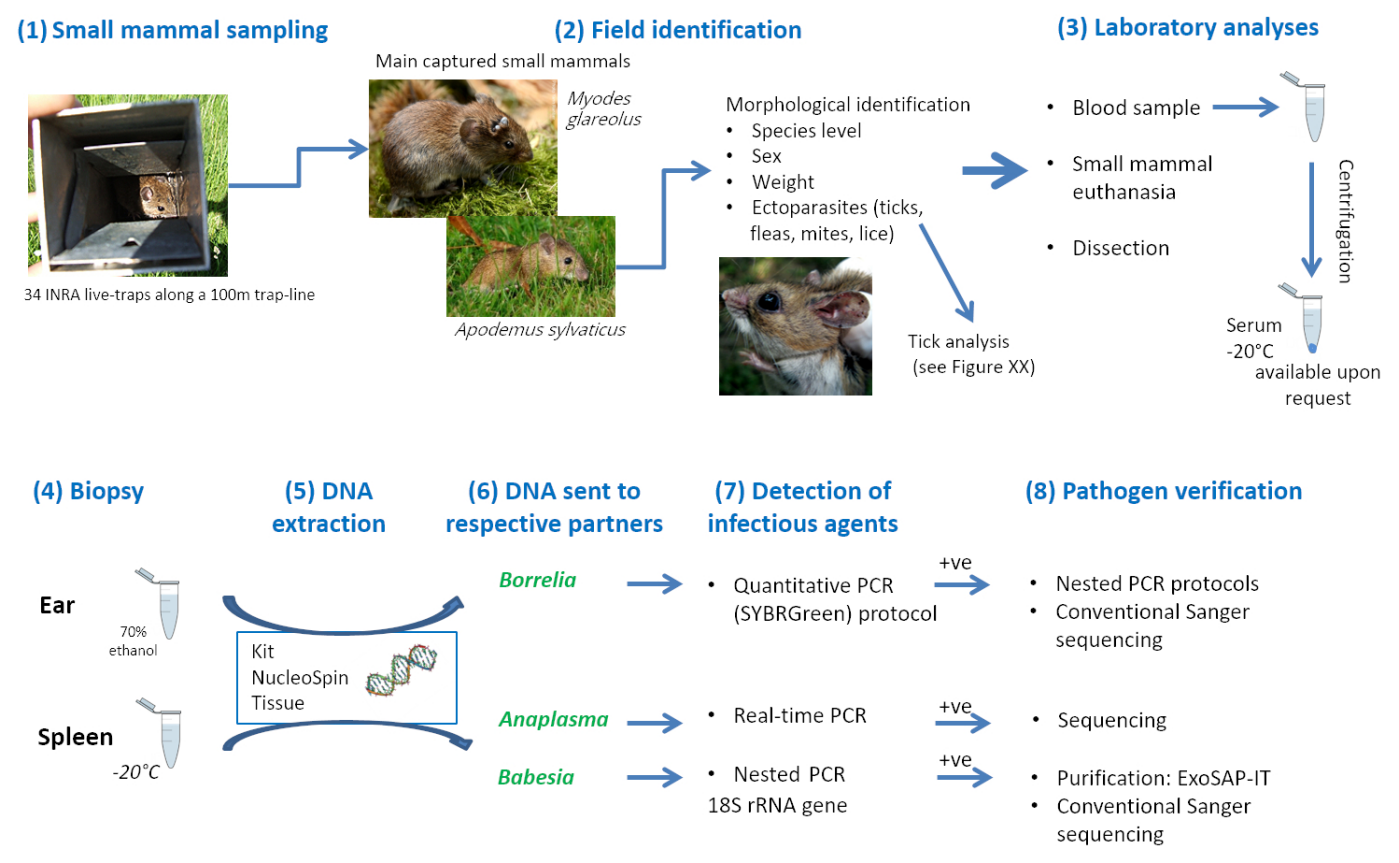
Molecular analyses of small mammals. +ve, positive sample.

The 100 m trap-line contained 34 INRAE live-traps, fitted with dormitory boxes and baited with a mixture of seeds and fresh apple. After placement, the traps were checked in the morning 24- and 48-hours after setup (Figs 3, 7). Captured small mammals were identified to species, sexed and weighed to 0.5 g in a field laboratory (Table 6). They were euthanised by authorised experimenters in accordance with French law and dissected. A blood sample and ear and spleen biopsies were performed for the detection and characterisation of infectious agents during the first four field campaigns. Blood sampling was performed on trapped animals using the retro-orbital method (Hoff 2000). Blood pellets were separated from serum by centrifugation. Serum samples were stored at −20°C and are available for supplementary analysis upon request. Ticks from small mammals were counted immediately after being euthanised in VG, but in ZA, due to the high number of captured mammals, dead animals were frozen and ticks were collected later during dissections. All collected ticks were stored in 70% ethanol for later identification and use for molecular analyses. The animals captured in spring 2014 were not euthanised, but were released at least 500 m away from the capture site to avoid recapture and ticks were quickly collected on these individuals.

***Molecular analyses***

- *In tick* (Fig. 6) (Table 7): Amongst the 12287 nymphs collected during the five campaigns, 4518 *I. ricinus* nymphs were selected at random from the two major periods of tick activity, i.e. spring campaigns of 2012 and 2013. For each tick, DNA was extracted using the ammonia-based protocol described in Schouls et al. (1999). *Borrelia* detection was performed using the quantitative PCR (SYBRGreen) protocol outlined in Jacquot et al. (2016). To identify the infecting *Borrelia* species, positive samples were re-amplified using nested PCR protocols for the FlaB and OspC genes (Gómez-Díaz et al. 2011) and amplicons were directly sequenced using Sanger technology (Eurofins, France). Detection of *A. phagocytophilum* DNA was ascertained by real-time PCR by targeting msp2/p44 genes and genotypes were characterised by 454 sequencing of groEL, msp4 and ankA genes (GATC, Germany) (Chastagner et al. 2017). The detection of *Babesia* spp. was achieved by nested PCR of the 18S rRNA gene (Jouglin et al. 2017). Positive amplicons were purified using ExoSAP-IT (Ozyme, France) and sent for Sanger sequencing (GATC, Germany). Additional investigations were also conducted on the population genetics of some ticks (nymphs), using either microsatellite (d'Ambrioso 2016) or SNP loci (Quillery et al. 2014).
- *In small mammals* (Fig. 7) (Tables 6, 8): Small mammals trapped in spring and autumn sessions of 2012 and 2013 were analysed for the three pathogenic agents (N = 300 small mammals in VG site and N = 608 in ZA site). However, a couple of individuals could not be tested for all pathogens because of insufficient DNA quantity. Spleens were stored at −20°C for detection of *A. phagocytophilum* (Chastagner et al. 2016) and *Babesia* (Jouglin et al. 2017). Ear biopsies were stored in 70% ethanol for detection of *Borrelia* spp. (Jacquot et al. 2016). DNA from spleen and ear samples were extracted using the NucleoSpinTissue kit (Macherey Nagel, Düren, Germany) (Chastagner et al. 2016, Perez et al. 2017). DNA of *A. phagocytophilum* was detected by real-time PCR targeting the msp2 gene, according to the protocol of Courtney et al. (2004). Detection of *Babesia* spp. was achieved by nested PCR of the 18S rRNA gene; different primers were used to amplify *Babesia* spp. from small mammals and from ticks because of high rates of false positive amplifications with small mammal DNA (Jouglin et al. 2017). Positive amplicons were purified using ExoSAP-IT (Ozyme, France) and sent for Sanger conventional sequencing (GATC, Germany). DNA of *B. burgdorferi* s.l. in ear samples was detected and typed as outlined for ticks.

***Livestock survey in VG site***

Livestock abundance was measured in the VG site on the pasture adjoining each tick transect-line in 2012 and 2013 (Table 9). The number of cattle, sheep, goats and horses grazing in each pasture was monitored on a weekly basis from autumn 2011 to spring 2013, excluding the winter (November to March). The number of individuals grazing in each pasture was then summed per season (spring: week 17 to 26, summer: week 27 to 35, autumn: week 36 to 44) to obtain a livestock abundance estimate, given as the number of head.day per season. When averaged per count day and summed across the whole VG site, the livestock mean density was 20.3 animals/km^2^ in the open landscapes (HH and LH).

***DataBase***

**Figure 8.**
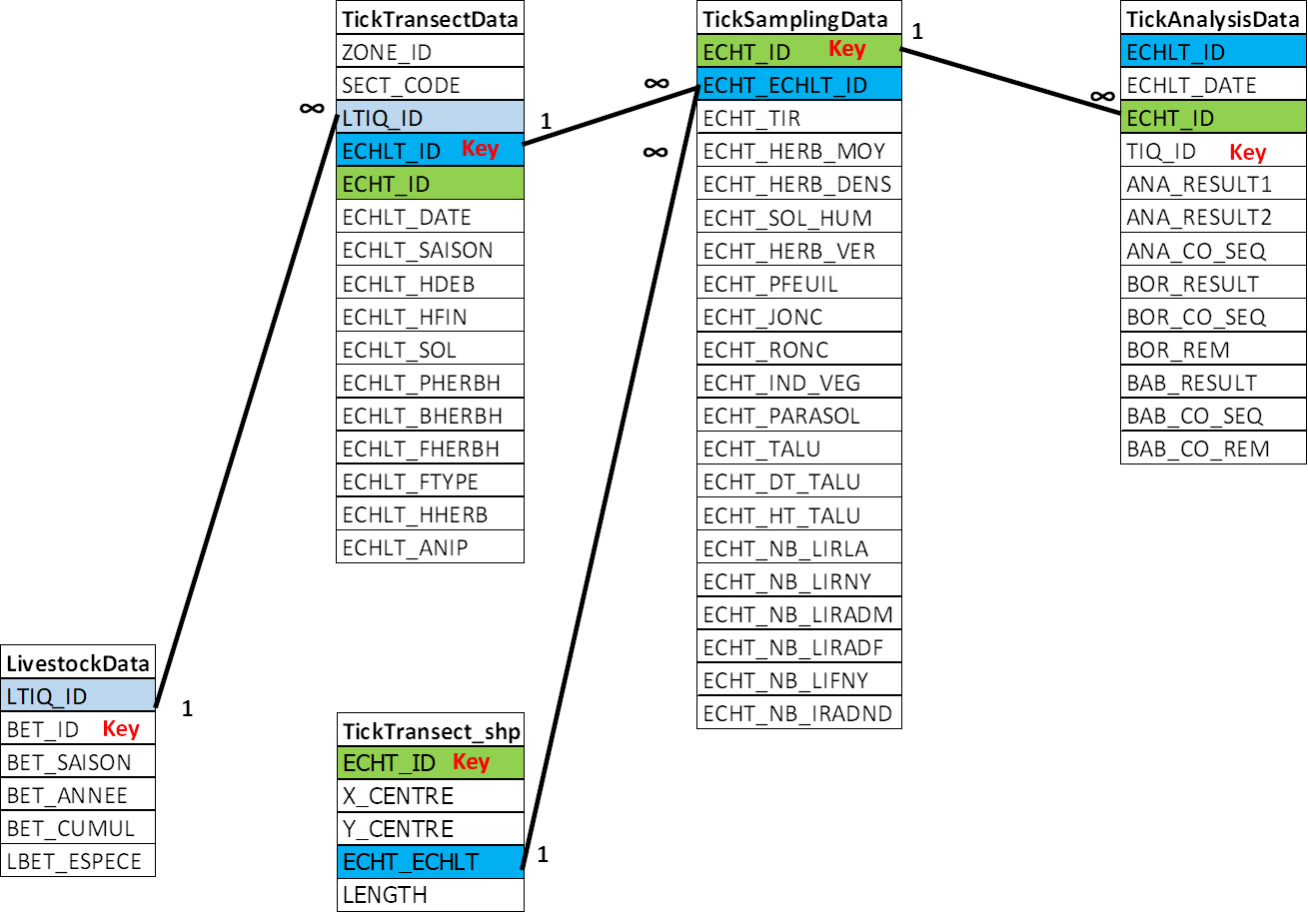
Relational model for ticks: relationships between tables concerning tick sampling and analyses. Similar colour corresponds to similar data present in two tables. Key is primary key. ECHLT_*, Identifier code for tick transect-line; ECHT_*, Identifier code for tick sub-transect line.

**Figure 9.**
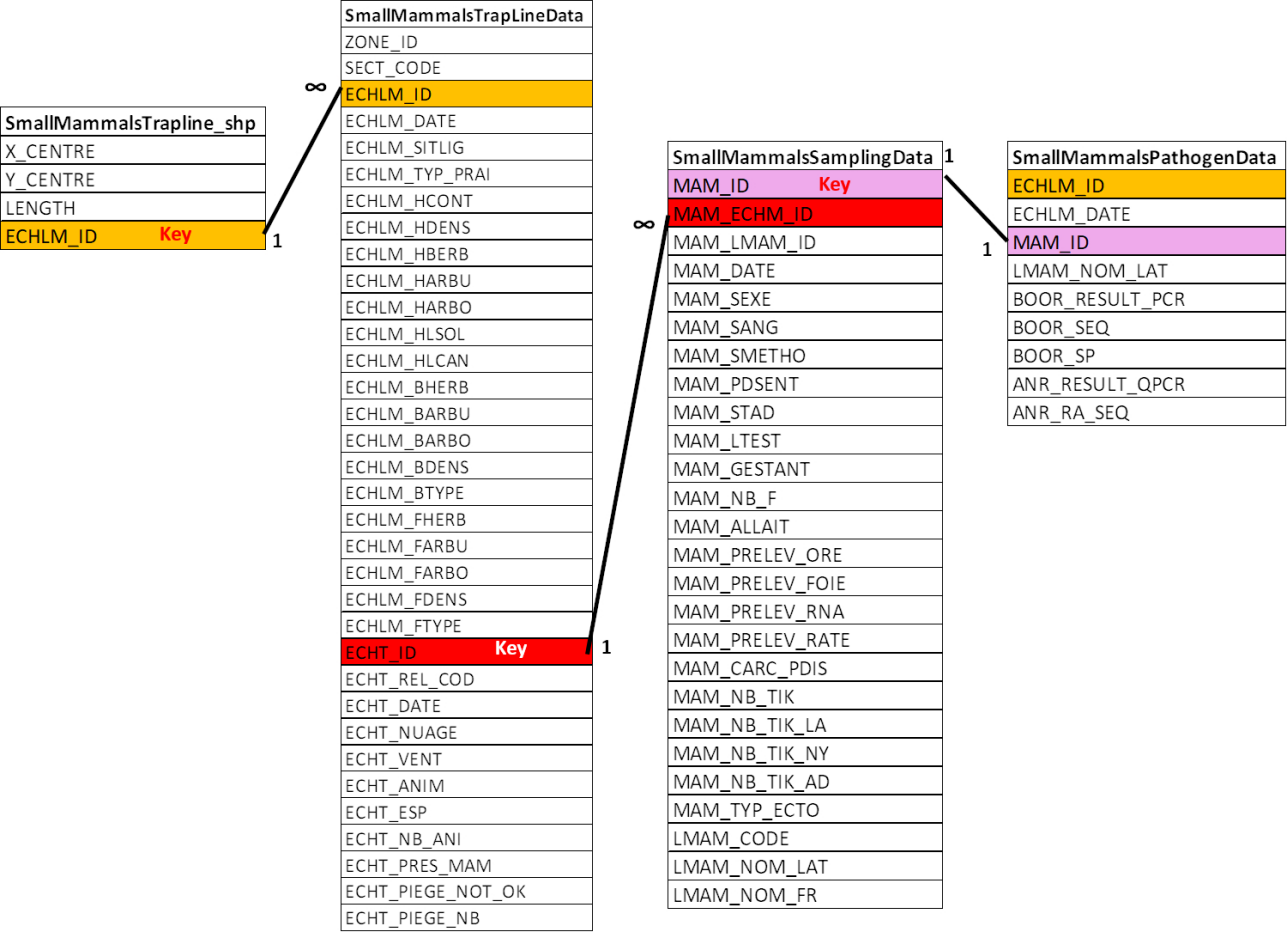
Relational model for small mammals: relationships between tables concerning small mammal sampling and analyses. Similar colour corresponds to similar data present in two tables. Key is primary key. ECHLM_*, Identifier code for small mammal trap-line; MAM_*, Identifier code for captured small mammal.

All the data of Tables 1, 2, 3, 4, 5, 6, 7, 8, 9 were united in a single Access database. The relationship between the tables is given in Figs 8, 9.

The data presented in this dataset are detailed by campaign and by site in Table 10.

***Variables not included in the datapaper***

Information on the infection rate and movement of roe deer in some of the studied habitat types were recorded at the VG site (see, for exemple, Martin et al. 2018). They are available on http://eurodeer.org/ or upon request to the CEFS.

Weather data were obtained from Météo-France weather stations close to ZA (Broualan, Rennes-St Jacques, Pontorson) and VG (Boussan, Fabas, Palaminy) sites. Additional weather data were measured near the VG site at the meteorological weather station (INRAE in SAMAN), located at the UMR DYNAFOR (INRAE-INPT) in Saint-André (F-31420) or near the ZA site at the COSTEL meteorological weather station (CNRS in COSTEL), located in the LEGT RENNES. According to the location, the weather stations were equipped with sensors to measure air and ground temperatures, air humidity, pluviometry, wind speed and direction, relative humidity, atmospheric pressure and light intensity. The data (2011-2014) are available upon request to the corresponding author.

Additional variables were calculated to measure landscape heterogeneity around the sampling locations. These data and their production (ecotone length between wooded habitat and meadows, proportion of woodland cover, grassland cover and crops, mean distance between wooded patches, perimeter-area ratio of wooded patches, connectivity of wooded habitat patch) are presented in Perez et al. (2016) and Perez et al. (2020).

## Geographic coverage

### Description

- VG site (19004 ha):

top left 43°22'11,59''N, 0°43'59,17''E;

bottom right: 43°11'41,25''N, 0°59'15,61''E

- ZA site (14203 ha):

top left 48°34'20,83''N, 1°19'21,26''W;

bottom right: 48°25'20,46''N, 1°29'56,85''W

## Usage rights

### Use license

Other

### IP rights notes

Creative Commons CC-BY 4.0

## Data resources

### Data package title

Data from ANR OSCAR Project

### Resource link

Portail Data INRAE, https://data.inrae.fr/

### Number of data sets

4

### Data set 1.

#### Data set name

Field description of tick datasets

#### Data format

tab

#### Number of columns

1

#### Character set

UTF-8

#### Download URL

https://data.inrae.fr/dataset.xhtml?persistentId=doi: 10.15454/93LPP7

#### Description

The data concerning questing tick sampling are presented in the 3 following tables.

Table 3. Field description of the dataset, including the characteristics of the questing tick transect-lines. (Associated file: TickTransectData.tab).

Table 4. Field description of the dataset, including characteristics of questing tick sampling in each tick sub-transect. (Associated file: TickSamplingData.tab).

Table 7. Field description of the dataset concerning the analyses of tick DNA for infectious agents. (Associated file: TickAnalysisData.tab).

The date format ISO 8601 (YYYY-MM-DD) was used.

### Data set 2.

#### Data set name

Description of small mammal datasets

#### Data format

tab

#### Number of columns

1

#### Character set

UTF-8

#### Download URL

https://data.inrae.fr/dataset.xhtml?persistentId=doi: 10.15454/93LPP7

#### Description

The data concerning small mammal sampling are presented in the 3 following tables.

Table 5. Field description of the characteristics of the small mammal trap-lines in the dataset. (Associated file: SmallMammalsTrapLineData.tab)

Table 6. Field description of the dataset concerning small mammal sampling and identification (Associated file: SmallMammalsSamplingData.tab)

Table 8. Field description of the dataset concerning the analyses of small mammal DNA for infectious agents (Associated file: SmallMammalsPathogenData.tab)

The date format ISO 8601 (YYYY-MM-DD) was used.

### Data set 3.

#### Data set name

Description of the livestock dataset

#### Data format

tab

#### Number of columns

1

#### Character set

UTF-8

#### Download URL

https://data.inrae.fr/dataset.xhtml?persistentId=doi: 10.15454/93LPP7

#### Description

Field description for the livestock dataset (Table 9) (Associated file: LivestockData.tab)

### Data set 4.

#### Data set name

Tick sub-transects and small mammal trap-line locations

#### Data format

shapefile

#### Number of columns

1

#### Download URL

https://data.inrae.fr/dataset.xhtml?persistentId=doi: 10.15454/93LPP7

#### Description

Two tables describing the sample locations for questing ticks (Table 1) and for small mammals (Table 2).

(Associated files: TickTransect.shp and SmallMammalsTrapLine.shp)

## Additional information

We provide a quick description of the results in the following section. A total of 29004 questing ticks and 1230 small mammals were collected during the study at the two sites and over the five campaigns. All questing nymphal (N = 12311) and adult ticks (646) were identified to species. Ticks from small mammals (N = 1359) were also identified to the stage.

### Sampled ticks

During the five campaigns (from spring 2012 to spring 2014), 16047 larvae, 12287 *I. ricinus* nymphs, 646 *I. ricinus* adults and 24 *Ixodes frontalis* nymphs were collected on the vegetation (Table 11).

**Figure 10.**
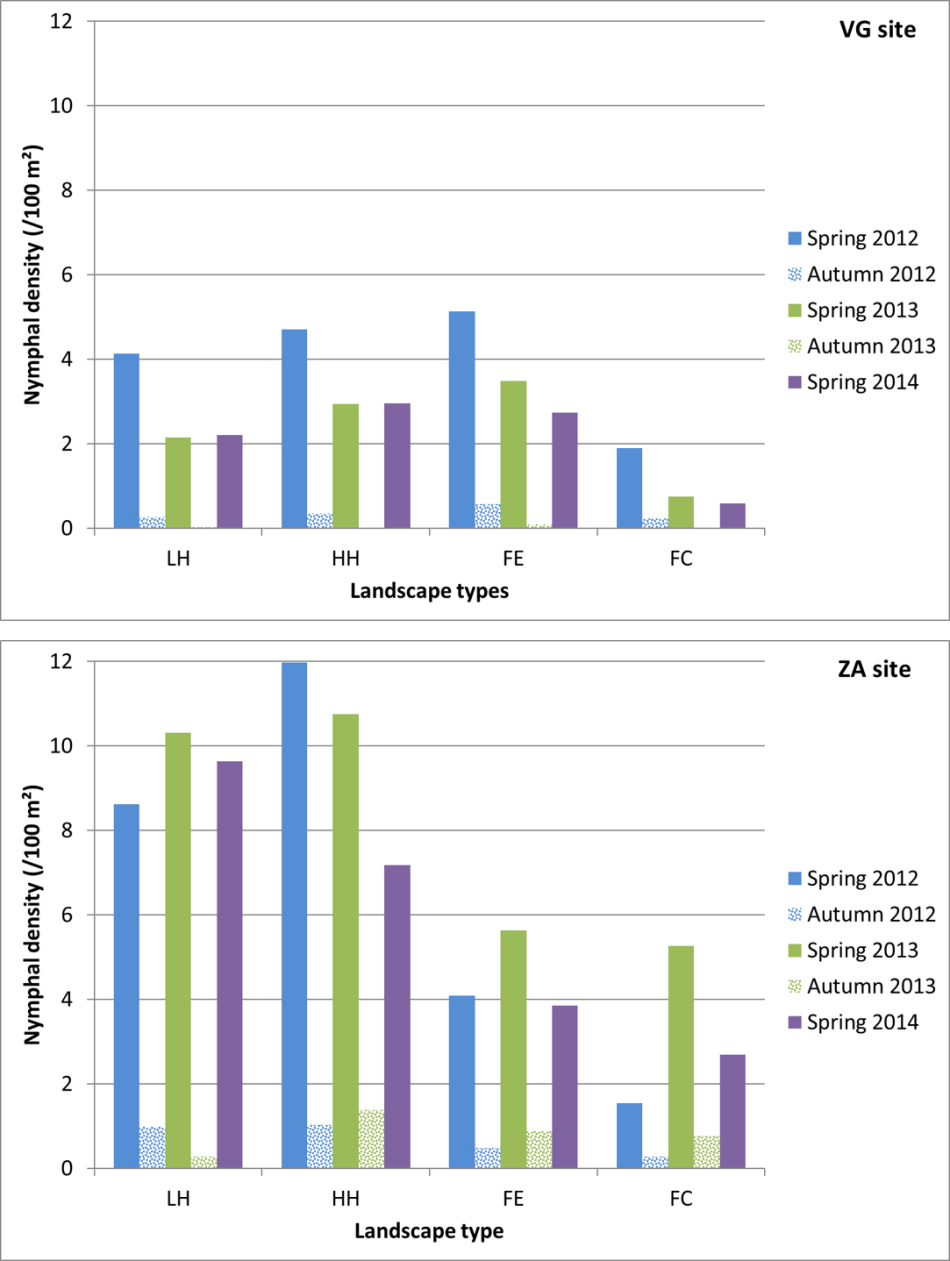
*I. ricinus* nymphal density in the two sites (VG and ZA), according to campaign and landscape type. Landscape types: LH: Agricultural landscapes with a Low Hedgerow network density HH: Agricultural landscapes with a High Hedgerow network density FE: Forest Edge FC: Forest Core

Fig. 10 presents the density of *I. ricinus* nymphs, according to landscape type and field campaign. Densities were generally higher in the ZA site than in the VG site, regardless of the campaign or landscape type. However, large heterogeneities were found amongst the five campaigns in both sites.

### Sampled small mammals

Over the study, 335 small mammals were trapped in the VG site (Table 12) and 895 in the ZA site (Table 13). Seven different species were found in VG against five in ZA. In both sites, wood mice (*Apodemus sylvaticus*) were the dominant species, accounting for 75% of the captured individuals. Bank vole (*Myodes glareolus*) was the second most frequently-encountered species in both sites (VG: 11% and ZA: 24%).

### Local environmental conditions

In the VG site, the forest type was mainly deciduous (N = 41) with one mixed forest (including coniferous trees). In the ZA site, collections were performed in 33 deciduous forest type and eight mixed forests. Table 14 presents some results of local environmental variables collected during tick sampling.

The livestock survey was performed in the VG site: livestock occurred on 28 of the 90 questing tick transect-lines, cattle being the main species present in pastures (Table 15). Median heads.day values at pasture was 112 for the 3 seasons (min = 0, max = 1848). Caprine were present along two transect-lines, equines along three transect-lines and ovine along three transect-lines. One meadow along a transect-line (VG-BD-L002) was occupied by the four livestock species.

### Pathogen results

A selected subset of questing nymphs (N = 4518 *I. ricinus*) and 908 trapped small mammals (N = 300 in VG site and N = 608 in ZA site) were analysed for the three pathogenic agents: *A. phagocytophilum*, *Borrelia* spp. and *Babesia* spp. (Table 16).

*Pathogen results in I. ricinus nymphs*. *A. phagocytophilum* was detected, respectively in 1.9% and 2.2% of questing *I. ricinus* nymphs from VG and ZA. Six species of *Borrelia* (*B. afzelii, B. burgdorferi sensu stricto, B. garinii, B. valaisiana, B. spielmani, B. turdi or B. lusitaniae*) were identified in nymphs in the two sites (Table 17). Amongst the 51 positive *I. ricinus* nymphs for *Babesia* spp. in the VG site, 23 were identified as *Babesia venatorum* and 11 had non-specific sequences. Amongst the 82 positive *I. ricinus* nymphs in the ZA site, 13 were identified as *B. venatorum*, two as *Babesia capreoli* and eight had non-specific sequences.

*Pathogen results in small mammals* (Table 16). *A. phagocytophilum* was not found in VG, but showed a prevalence of 6.9% in small mammals of ZA (Chastagner et al. 2016). Small mammals were infected only by *B. afzelii* with respective prevalences of 4.2% and 4.1% in VG and ZA. Amongst the six small mammals infected by *Borrelia* in the VG site, five were *A. sylvaticus* and one was *M. glareolus*. In the ZA site, amongst the 26 infected small mammals, 14 were *A. sylvaticus*, 11 were *M. glareolus* and one *Microtus subterraneus* (Perez et al. 2017). In the VG site, small mammals were not screened for *Babesia* spp. In the ZA site, one small mammal (*M. glareolus*, 2-ZA-CF-LM092-M3) amongst 597 tested was positive for *Babesia* (Jouglin et al. 2017).

## Figures and Tables

**Figure 4a.**
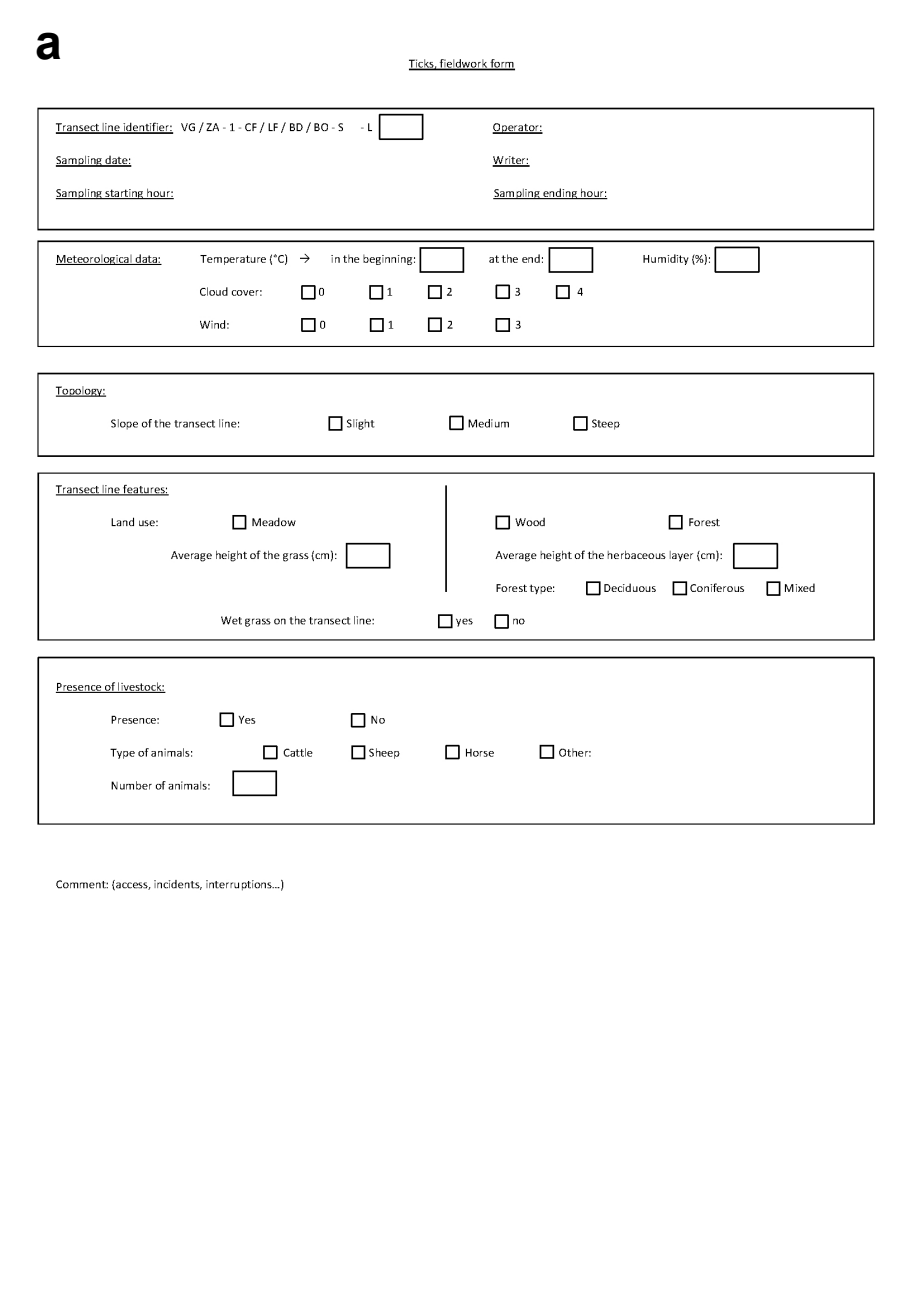
Page 1

**Figure 4b.**
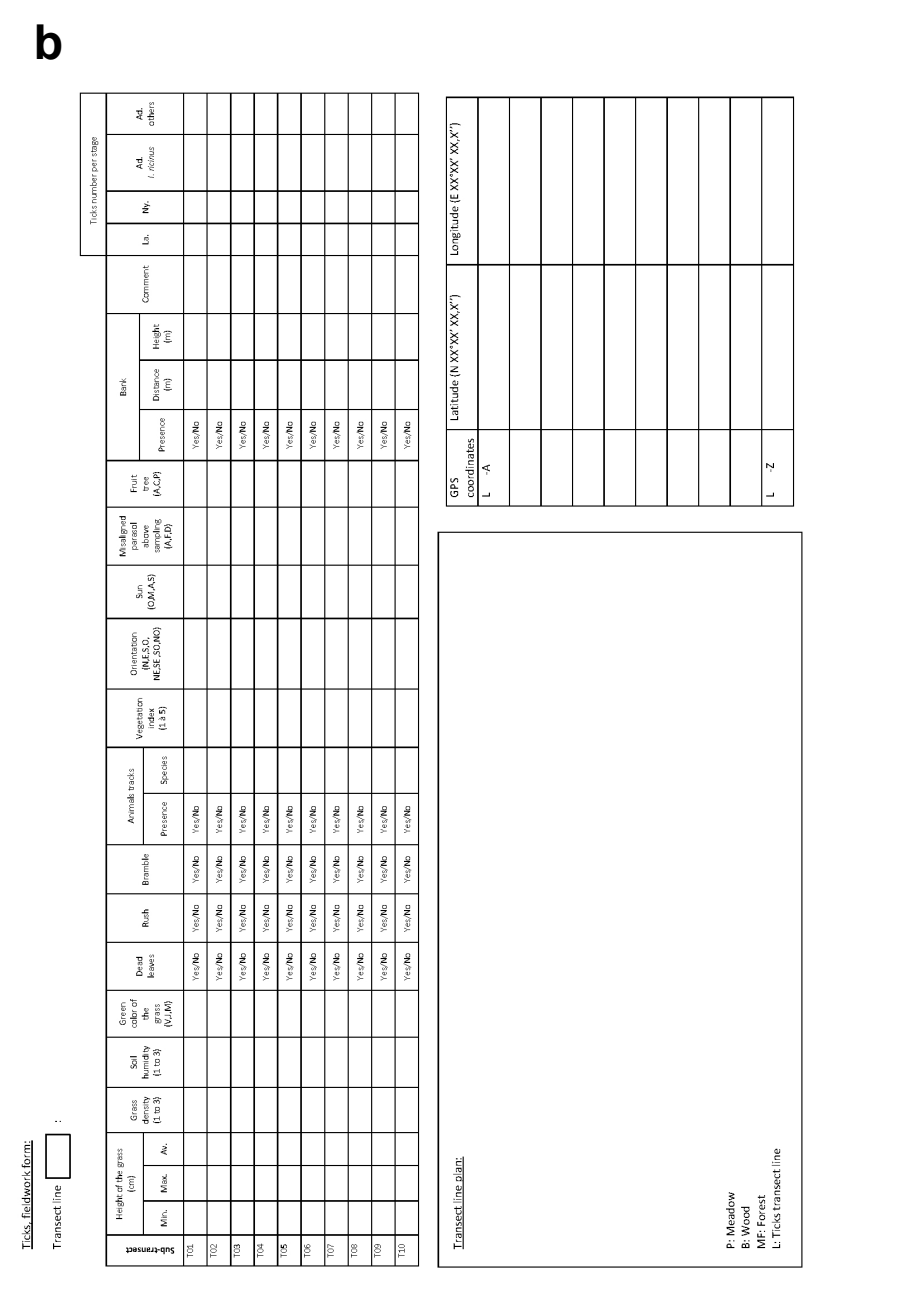
Page 2

**Figure 5a.**
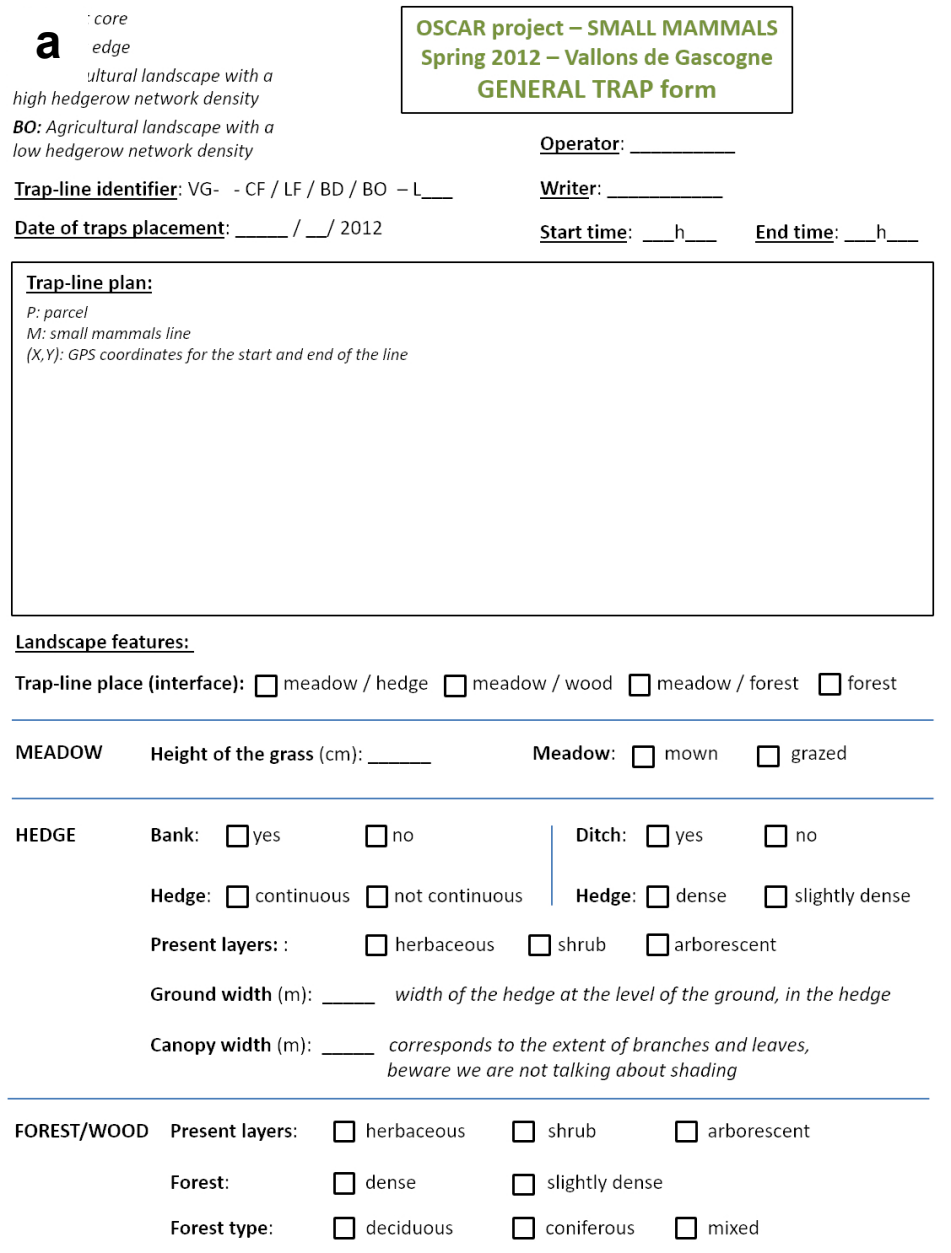
Page 1

**Figure 5b.**
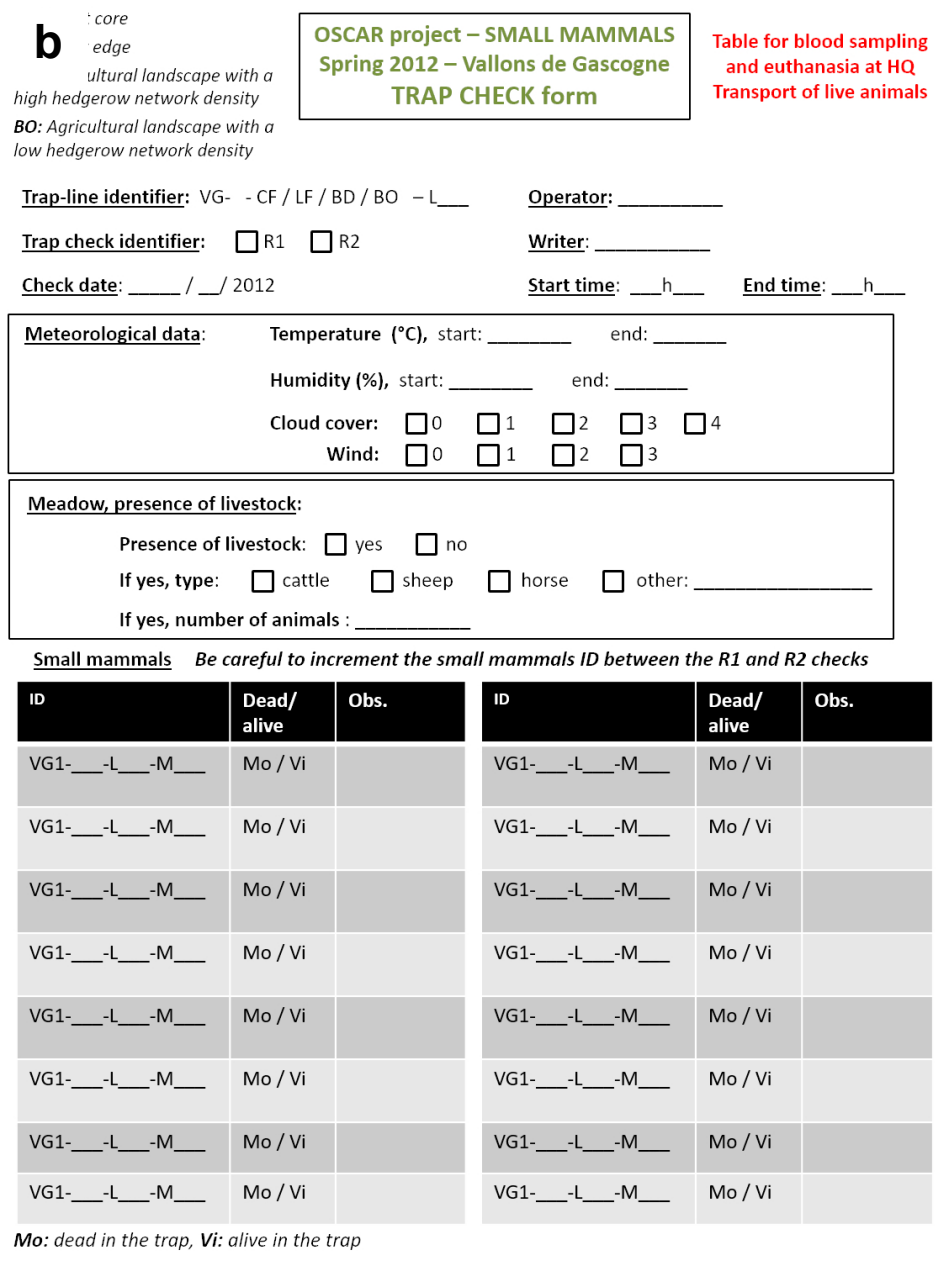
Page 2

**Figure 5c.**
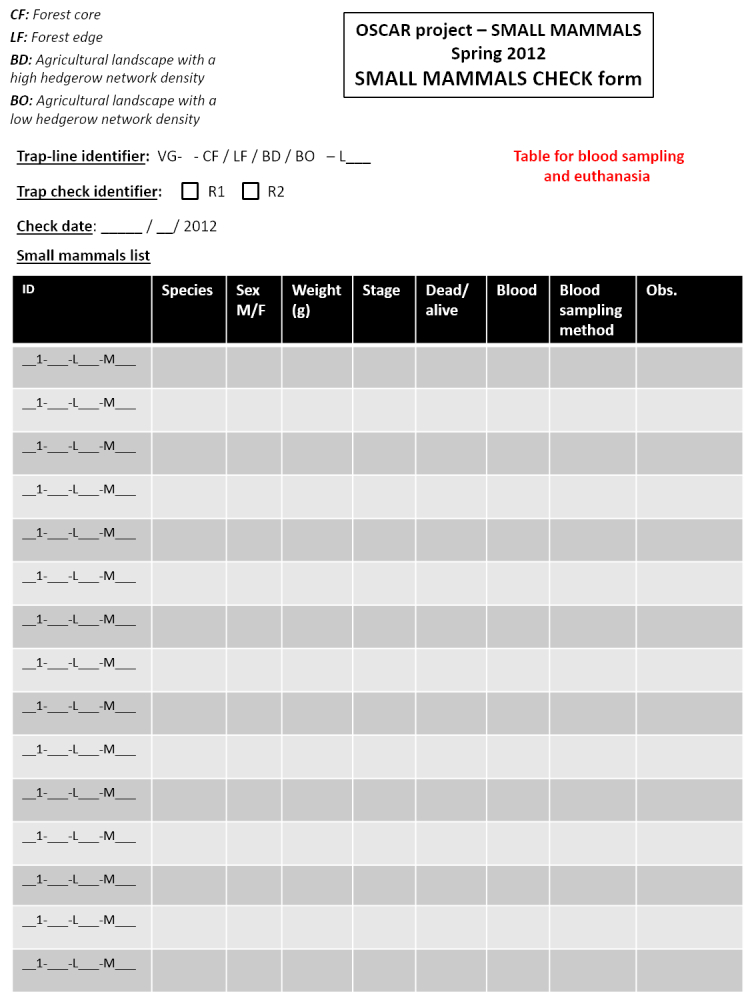
Page 3

**Table 1.** Field description for tick sub-transect locations. c., characters.

**Field**	**Description**	**Type**
ECHT_ID	Identifier for tick sub-transect line: campaign - site - landscape type - transect line number - sub-transect line number	Text (50 c.)
X_CENTRE	X coordinate of the sub-transect centroid (RGF93_Lambert_93, EPSG 2154)	Real (19, 11)
Y_CENTRE	Y coordinate of the sub-transect centroid (RGF93_Lambert_93, EPSG 2154)	Real (19, 11)
ECHT_ECHLT	Identifier for the transect: campaign - site -landscape type - transect line number	Text (50 c.)
LENGTH	Length of the sub-transect (metres)	Real (13, 11)
LATITUDE	Decimal Latitude of the sub-transect centroid (WGS84; EPSG 4326)	Real (10, 7)
LONGITUDE	Decimal Longitude of the sub-transect centroid (WGS84; EPSG 4326)	Real (10, 7)

**Table 2.** Field description for small mammal trap-line locations. c., characters.

**Field**	**Description**	**Type**
X_CENTRE	X coordinate of the trap-line centroid (RGF93_Lambert_93, EPSG 2154)	Real (18, 11)
Y_CENTRE	Y coordinate of the trap-line centroid (RGF93_Lambert_93, EPSG 2154)	Real (18, 11)
LENGTH	Length of the trap-line (metres)	Real (12, 11)
ECHLM_ID	Identifier of the trap-line: campaign - site - landscape type - trap-line number	Text (15 c.)
LATITUDE	Decimal Latitude of the sub-transect centroid (WGS84; EPSG 4326)	Real (10, 7)
LONGITUDE	Decimal Longitude of the sub-transect centroid (WGS84; EPSG 4326)	Real (10, 7)

**Table 3.** Field description of the dataset including the characteristics of the tick transect lines. c., characters.

**Field**	**Description**	**Type**
ZONE_ID	Identifier of the LTER site (VG or ZA)	Text (5 c.)
SECT_CODE	Identifier for the landscape type: forest core (FC, CF in table), forest edge (FE, LF in table), agricultural landscape with a high hedgerow network density (HH, BD in table), agricultural landscape with a low hedgerow network density (LH, BO in table)	Text (5 c.)
LTIQ_ID	Identifier for the transect line: site - landscape type - transect line number	Text (20 c.)
ECHLT_ID	Identifier for the transect line: campaign - site - landscape type - transect line number	Text (20 c.)
ECHT_ID	Identifier for tick sub-transect line: campaign - site - landscape type - transect line number - sub-transect line number	Text (30 c.)
ECHLT_DATE	Sampling date for a transect	Date/Time
ECHLT_SAISON	Identifier for campaign (1 = spring 2012, 2 = autumn 2012, 3 = spring 2013, 4 = autumn 2013, 5 = spring 2014)	Integer
ECHLT_HDEB	Starting hour of tick sampling in the transect	Date/Time
ECHLT_HFIN	Ending hour of tick sampling in the transect	Date/Time
ECHLT_SOL	Land use: 1 = meadow, 2 = wood, 3 = forest, 4 = meadow/hedge, 5 = meadow/wood, 6 = meadow/forest	Boolean
ECHLT_PHERBH	Average height of the grass in the meadow landscape (cm)	Integer
ECHLT_BHERBH	Average height of the grass in the wood landscape (cm)	Integer
ECHLT_FHERBH	Average height of the grass in the forest landscape (cm)	Integer
ECHLT_FTYPE	Forest type: 1 = deciduous, 2 = coniferous, 3 = mixed	Boolean
ECHLT_HHERB	Wet grass: 1 = yes, 0 = no	Boolean
ECHLT_ANIP	Presence of livestock on the pasture: 1 = yes, 0 = no	Boolean

**Table 4.** Field description of the dataset including characteristics of tick sampling in each tick sub-transect. c., characters.

**Field**	**Description of the sub-transect**	**Type**
ECHT_ID	Identifier for the tick sub-transect	Text (30 c.)
ECHT_ECHLT_ID	Key to Table 3	Text (20 c.)
ECHT_TIR	Identifier of sub-transect	Text (3 c.)
ECHT_HERB_MOY	Average height of the grass in the sub-transect (cm)	Boolean
ECHT_HERB_DENS	Grass in the sub-transect: 1 = none, 2 = sparse, 3 = dense	Boolean
ECHT_SOL_HUM	Soil humidity: 1 = dry, 2 = slightly wet, 3 = presence of water	Real
ECHT_HERB_VER	Green colour of the grass: V = green on 2/3 of the sub-transect, J = yellow on 2/3 of the sub-transect, M = mixed, NP = not relevant if no grass	Text (3 c.)
ECHT_PFEUIL	Presence of dead leaves: 1 = yes, 0 = no	Boolean
ECHT_JONC	Presence of rush: 1 = yes, 0 = no	Boolean
ECHT_RONC	Presence of bramble: 1 = yes, 0 = no	Boolean
ECHT_IND_VEG	Vegetation index (hedge or wood): 1 = no hedge, 2 = discontinuous hedge, 3 = continuous hedge not deeper than 2 m, 4 = deeper hedge, between 2 and 5 m, 5 = hedge deeper than 5 m or wood	Boolean
ECHT_PARASOL	Misaligned parasol above sampling: A = no branches (no parasol), F = dense branches over less than 2/3 of the sub-transect, D = dense branches over more than 2/3 of the sub-transect	Text (1 c.)
ECHT_TALU	Presence of a bank: 1 = yes, 0 = no	Boolean
ECHT_DT_TALU	Distance between the bank and the sub-transect (metres)	Real
ECHT_HT_TALU	Bank height (metres)	Real
ECHT_NB_LIRLA	Number of *Ixodes ricinus* larvae	Boolean
ECHT_NB_LIRNY	Number of *Ixodes ricinus* nymphs	Boolean
ECHT_NB_LIRADM	Number of *Ixodes ricinus* male adults	Boolean
ECHT_NB_LIRADF	Number of *Ixodes ricinus* female adults	Boolean
ECHT_NB_LIFNY	Number of *Ixodes frontalis* nymphs	Boolean
ECHT_NB_IRADND	Number of adult *Ixodes ricinus* ticks (male or female)	Boolean

**Table 5.** Field description of the dataset including characteristics of the small mammal trap-lines. c., characters.

**Field**	**Description**	**Type**
ZONE_ID	Identifier of the LTER site (VG or ZA)	Text (5 c.)
SECT_CODE	Identifier of the landscape type: forest core (FC, CF in table), forest edge (FE, LF in table), landscape with high hedgerow network density (HH, BD in table), landscape with low hedgerow network density (LH, BO in table)	Text (5 c.)
ECHLM_ID	Identifier of the trap-line: campaign - site - landscape type - trap-line number	Text (30 c.)
ECHLM_DATE	Sampling date for placing the traps	Date/Time
ECHLM_SITLIG	Trap-line place (interface): 1 = meadow/hedge, 2 = meadow/wood, 3 = meadow/forest, 4 = forest	Boolean
ECHLM_TYP_PRAI	Meadow type: 1 = grasses, 2 = mowing meadow, 3 = other	Boolean
ECHLM_HCONT	Continuity of the hedge: 1 = continuous, 2 = not continuous	Boolean
ECHLM_HDENS	Hedge density: 1 = dense, 2 = slightly dense	Boolean
ECHLM_HBERB	Presence of herbaceous layer in hedge: 1 = yes, 0 = no	Boolean
ECHLM_HARBU	Presence of shrub layer in hedge: 1 = yes, 0 = no	Boolean
ECHLM_HARBO	Presence of arborescent layer in hedge: 1 = yes, 0 = no	Boolean
ECHLM_HLSOL	Width of the hedge at the level of the ground, in the hedge (metres)	Integer
ECHLM_HLCAN	Width of the canopy above the hedge (metres)	Boolean
ECHLM_BHERB	Presence of a herbaceous layer in the woods: 1 = yes, 0 = no	Boolean
ECHLM_BARBU	Presence of shrub layer in the woods: 1 = yes, 0 = no	Boolean
ECHLM_BARBO	Presence of arborescent layer in the woods: 1 = yes, 0 = no	Boolean
ECHLM_BDENS	Wood density: 1 = dense, 2 = slightly dense	Boolean
ECHLM_BTYPE	Wood type: 1 = deciduous, 2 = coniferous, 3 = mixed	Boolean
ECHLM_FHERB	Presence of herbaceous layer in forest: 1 = yes, 0 = no	Boolean
ECHLM_FARBU	Presence of shrub layer in forest: 1 = yes, 0 = no	Boolean
ECHLM_FARBO	Presence of arborescent layer in forest: 1 = yes, 0 = no	Boolean
ECHLM_FDENS	Forest density: 1 = dense, 2= slightly dense	Boolean
ECHLM_FTYPE	Forest type: 1 = deciduous, 2 = coniferous, 3 = mixed	Boolean
ECHT_ID	Identifier for small mammal trap-line and checking number	Text (30 c.)
ECHT_REL_COD	Identifier of trap checks: R1 = 24 h, R2 = 48 h	Text (5 c.)
ECHT_DATE	Day of trap check	Date/Time
ECHT_NUAGE	Cloud cover: 0 = blue sky, 1 = 1/4 cloud cover, 2 = half covered, 3 = 3/4 covered, 4 = completely covered	Integer
ECHT_VENT	Presence of wind: 0= no wind, 1 = light wind, 2 = discontinuous, 3 = strong	Boolean
ECHT_ANIM	Presence of livestock in the field: 1 = yes, 0 = no	Boolean
ECHT_ESP	Animal types: 1 = cattle, 2 = sheep, 3 = horse, 4 = other	Boolean
ECHT_NB_ANI	Number of animals in the field	Boolean
ECHT_PRES_MAM	Small mammal sign: 1 = yes, 0 = no	Boolean
ECHT_PIEGE_NOT_OK	Traps disturbed or closed without capture: 1 = yes, 0 = no	Boolean
ECHT_PIEGE_NB	Number of traps disturbed or closed without capture (between 1 and 34)	Integer

**Table 6.** Field description of the dataset concerning small mammal sampling and identification. c., characters.

**Field**	**Description**	**Type**
MAM_ID	Identifier of the trapped small mammals: campaign - site - landscape type - trap-line number - small mammal number	Text (30 c.)
MAM_ECHM_ID	Identifier for small mammal trap-line and check number	Text (30 c.)
MAM_DATE	Autopsy day	Date
MAM_SEXE	Identifier for sex: 1 = Male, 2 = Female	Boolean
MAM_SANG	Blood sampling: 1 = yes, 0 = no	Boolean
MAM_SMETHO	Blood sampling method: IC = intracardiac, RO = retro-orbital	Text (2 c.)
MAM_PDSENT	Small mammal weight before autopsy (g)	Integer
MAM_STAD	Small mammal stage: 1 = juvenile, 2 = sub-young, 3 = adult	Boolean
MAM_LTEST	Testicule length	Boolean
MAM_GESTANT	Pregnant female: 1 = yes, 0 = no	Boolean
MAM_NB_F	If pregnant = yes, number of fœtuses	Boolean
MAM_ALLAIT	Lactating female: 1 = yes, 0 = no	Boolean
MAM_PRELEV_ORE	Ear sample: 1 = yes, 0 = no	Boolean
MAM_PRELEV_FOIE	Liver sample: 1 = yes, 0= no	Boolean
MAM_PRELEV_RNA	RNA sample from spleen: 1 = yes, 0 = no	Boolean
MAM_PRELEV_RATE	Spleen sample: 1 = yes, 0 = no	Boolean
MAM_CARC_PDIS	Carcass partially dissected and frozen: 1 = yes, 0 = no	Boolean
MAM_NB_TIK	Total number of ticks on the small mammal	Boolean
MAM_NB_TIK_LA	Total number of larvae on the small mammal	Boolean
MAM_NB_TIK_NY	Total number of nymphs on the small mammal	Boolean
MAM_NB_TIK_AD	Total number of adult ticks on the small mammal	Boolean
MAM_TYP_ECTO	Ectoparasitic species: fleas, mites, lice, fleas + mites, fleas + lice, mites + lice, fleas + mites + lice, ectoparasite species not specified, none	Text (50 c.)
LMAM_NOM_LAT	Species name (Latin)	Text (50 c.)
LMAM_NOM_FR	Species name (French)	Text (50 c.)
MAM_ID	Identifier of the trapped small mammals: campaign - site - landscape type - trap-line number - small mammal number	Text (30 c.)
MAM_ECHM_ID	Identifier for small mammal trap-line and check number	Text (30 c.)

**Table 7.** Field description of the dataset concerning the analyses of tick DNA for infectious agents. c.: characters

**Field**	**Description**	**Type**
ECHLT_ID	Identifier of the transect: season-site-landscape-transect number - Identifier for campaign (1 = spring 2012, 3 = spring 2013)	Text (20 c.)
ECHLT_DATE	Sampling date for a transect	Date/Time
ECHT_ID	Identifier for the tick transect -subtransect: campaign - site - landscape - transect number - sub-transect number	Text (30 c.)
TIQ_ID	Identifier for a tick	Text (30 c.)
ANA_RESULT1	Result method 1: detection of *Anaplasma* from tick DNA (yes = 1, no = 0)	Boolean
ANA_RESULT2	Result method 2: detection of *Anaplasma* from tick DNA (yes = 1, no = 0)	Boolean
ANA_CO_SEQ	Sequencing analysis: obtained sequence for *Anaplasma* (yes = 1, no = 0)	Boolean
BOR_RESULT	Result: detection of *Borrelia* from tick DNA (yes = 1, no = 0)	Boolean
BOR_CO_SEQ	Sequencing analysis: obtained sequence for *Borrelia* (yes = 1, no = 0)	Boolean
BOR_REM	Remark: assignment to a species	Memo
BAB_RESULT	Result: detection of *Babesia* by PCR from tick DNA (yes = 1, no = 0)	Boolean
BAB_CO_SEQ	Sequencing analysis: obtained sequence for *Babesia* (yes = 1, no = 0)	Integer
BAB_CO_REM	Remark: assignment to a species	Memo

**Table 8.** Field description of the dataset concerning the analyses of infectious agents from small mammals. c.: characters.

**Field**	**Description**	**Type**
ECHLM_ID	Identifier of the trap-line: campaign - site - landscape type - trap-line number	Text (30 c.)
ECHLM_DATE	Sampling date for the placement of traps	Date/Time
MAM_ID	Identifier of the trapped small mammals: campaign - site - landscape type - trap-line number - small mammal number	Text (30 c.)
LMAM_NOM_LAT	Species name	Text (50 c.)
BOOR_RESULT_PCR	Result: detection of *Borrelia* from small mammal ear DNA: 1 = yes, 0 = no	Boolean
BOOR_SEQ	Sequencing analysis of *Borrelia*: 1 = yes, 0 = no	Boolean
BOOR_SP	Species name of *Borrelia*	Memo
ANR_RESULT_QPCR	Result: detection of *Anaplasma* from spleen DNA: 1 = yes, 0 = no	Boolean
ANR_RA_SEQ	Sequencing analysis: obtained sequence for *Anaplasma* (1 = yes, 0 = no)	Integer

**Table 9.** Field description for livestock dataset. c., characters. Heads.day refers to the number of individual animals that were counted in a pasture on a given day.

**Field**	**Description**	**Type**
LTIQ_ID	Identifier for the transect line: site - landscape type - transect line number	Text (20 c.)
BET_ID	Identifier for livestock	Text (30 c.)
BET_SAISON	Season: spring (week 17 to 26), summer (week 27 to 35), autumn (week 36 to 44)	Text (10 c.)
BET_ANNEE	Year	Integer
BET_CUMUL	Sum of livestock heads.day at pasture over the considered season (spring 70 days, summer 63 days, autumn 63 days)	Integer
LBET_ESPECE	Species name: bovine, caprine, equine, ovine	Text (20 c.)

**Table 10.** Summary of available data in the present dataset according to campaign and site. Identifier for campaigns: 1 = spring 2012, 2 = autumn 2012, 3 = spring 2013, 4 = autumn 2013, 5 = spring 2014.

**Site**	**VG**					**ZA**				
**Campaign**	**1**	**2**	**3**	**4**	**5**	**1**	**2**	**3**	**4**	**5**
Local environmental conditions	yes	yes	yes	yes	yes	yes	yes	yes	yes	yes
Number of tick transect lines	90	90	90	36	90	89	89	90	36	90
Tick identification	yes	yes	yes	yes	yes	yes	yes	yes	yes	yes
Pathogens analysis in ticks	yes	no	yes	no	no	yes	no	yes	no	no
Number of small mammal trap-lines	24	24	24	24	24	24	24	24	24	24
Small mammal identification	yes	yes	yes	yes	yes	yes	yes	yes	yes	yes
Pathogens analysis in small mammals	yes	yes	yes	yes	no	yes	yes	yes	yes	no
Identification of small mammals ticks	yes	yes	yes	yes	no	yes	yes	yes	yes	no
Livestock	yes	yes	yes	yes	no	no	no	no	no	no

**Table 11.** Number of collected ticks per campaign and per site. No, number; IR, *Ixodes ricinus*; IF, *Ixodes frontalis*. Identifier for campaigns: 1 = spring 2012, 2 = autumn 2012, 3 = spring 2013, 4 = autumn 2013, 5 = spring 2014.

**Campaign**	**Site**	**No sampled transect-lines**	**No larvae**	**No IR nymphs**	**No IR adults**	**No IF nymphs**
1	VG	90	24	1588	59	1
1	ZA	89	5214	2622	109	7
2	VG	90	758	143	11	0
2	ZA	89	3649	277	22	7
3	VG	90	69	932	85	0
3	ZA	90	1508	3196	164	0
4	VG	36	27	16	8	0
4	ZA	36	867	330	20	4
5	VG	90	25	848	69	0
5	ZA	90	3906	2335	99	5
		**Total**	**16047**	**12287**	**646**	**24**

**Table 12.** Small mammal species in the VG site over the 5 field campaigns

**Species name**	**Number of captured individuals**
*Apodemus sylvaticus*	250
*Myodes glareolus*	37
*Crocidura russula*	18
*Microtus arvalis*	14
*Sorex coronatus*	11
*Microtus agrestis*	4
*Microtus pyrenaicus*	1
**Total**	**335**

**Table 13.** Small mammal species in the ZA site over the 5 field campaigns.

**Species name**	**Number of captured individuals**
*Apodemus sylvaticus*	668
*Myodes glareolus*	216
*Microtus agrestis*	4
*Sorex coronatus*	4
*Microtus subterraneus*	3
**Total**	**895**

**Table 14.** Summary values of local environmental conditions for transects and sub-transects in VG and ZA sites for the 5 field campaigns (1 to 5). Description of the fields are given in Tables 3, 4. NC: Not concerned (The field makes no sense for the landscape type in question. For example, there cannot be information in a field concerning meadows when the sub-transect line is in the forest); ND: Not documented (missing data).

**Transects and sub-transects**	**Site**	**VG**					**ZA**				
	**Campaign**	**1**	**2**	**3**	**4**	**5**	**1**	**2**	**3**	**4**	**5**
Number of tick transect lines		**90**	**90**	**90**	**36**	**90**	**89**	**89**	**90**	**36**	**90**
ECHLT_PHERBH	Median	20	10	50	20	30	45	20	30	13,5	60
	Min	5	5	15	10	5	10	10	10	0	0
	Max	60	120	105	50	50	110	50	160	100	110
ECHLT_BHERBH	Median	20	15	30	20	30	20	10	10	7,5	20
	Min	5	0	5	5	10	0	5	0	5	0
	Max	40	35	60	40	50	80	100	30	15	100
ECHLT_FHERBH	Median	20	25	30	22,5	25	15	17,5	15	10	20
	Min	0	5	15	5	10	5	5	0	0	5
	Max	30	30	55	40	60	20	20	50	30	30
Number of sub-transect		900	900	900	360	900	890	890	900	900	900
ECHT_HERB_DENS	1	172	161	93	83	64	291	293	254	117	176
	2	304	311	282	105	178	193	129	226	97	231
	3	424	428	524	172	657	404	468	420	146	492
	ND	0	0	1	0	1	2	0	0	0	1
ECHT_SOL_HUM	1	282	721	133	344	189	684	807	685	331	731
	2	514	141	665	15	634	156	71	195	27	154
	3	104	38	101	0	76	35	12	20	2	14
	ND	0	0	1	1	1	15	0	0	0	1
ECHT_HERB_VER	J	31	224	2	43	0	0	78	61	15	23
	M	147	339	15	59	54	7	134	89	64	34
	ND	0	0	1	0	1	14	0	0	0	1
	NC	3	14	24	26	1	79	92	103	89	63
	V	719	323	858	232	844	790	586	647	192	779
ECHT_PFEUIL	0	321	171	433	108	318	385	404	388	118	497
	1	579	729	467	252	581	488	473	512	242	403
	ND	0	0	0	0	1	17	13	0	0	0
ECHT_JONC	0	878	887	892	354	879	809	789	798	327	761
	1	22	13	7	5	20	59	90	102	33	139
	ND	0	0	1	1	1	22	11	0	0	0
ECHT_RONC	0	679	627	544	169	574	669	571	684	265	659
	1	211	273	353	190	322	200	289	214	94	241
	ND	10		3	1	4	21	30	2	1	0
ECHT_IND_VEG	1	6	7	7	1	7	22	13	23	1	16
	2	65	69	23	5	22	101	66	99	17	72
	3	37	75	53	15	27	62	62	108	46	112
	4	119	73	98	21	83	68	75	37	8	31
	5	603	596	715	317	679	637	663	633	288	668
	ND	70	80	4	1	82	0	11	0	0	1
ECHT_PARASOL	A	247	258	255	82	244	122	151	210	47	123
	D	387	383	483	207	173	327	412	465	205	530
	F	266	119	162	71	201	370	224	225	104	246
	ND	0	140	0	0	282	71	103	0	4	1
ECHT_TALU	0	819	817	828	349	774	698	526	561	169	420
	1	81	82	70	10	126	191	295	338	189	477
	ND	0	1	2	1	0	1	69	1	2	3

**Table 15.** Results of livestock survey in the VG site: sum of heads.day by species at pasture over the considered season (spring = 70 days, summer = 63 days, autumn = 63 days). Transect name (site - landscape type - transect number). Identifier for the landscape type: BD (bocage dense) = agricultural landscape with a high hedgerow network density (HH), BO (bocage ouvert) = agricultural landscape with a low hedgerow network density (LH), LF (Lisière de forêt) = forest edge (FE)

**Livestock**	**Transect name**	**Spring**	**Summer**	**Autumn**	**Total**
bovine	VG-BD-L002	0	322	413	**735**
	VG-BD-L004	0	0	56	**56**
	VG-BD-L006	420	378	378	**1176**
	VG-BD-L015	0	0	168	**168**
	VG-BD-L020	0	0	112	**112**
	VG-BD-L032	420	378	378	**1176**
	VG-BD-L033	0	546	364	**910**
	VG-BD-L034	0	567	637	**1204**
	VG-BD-L035	112	168	77	**357**
	VG-BD-L036	0	126	0	**126**
	VG-BD-L044	56	224	56	**336**
	VG-BD-L046	0	21	224	**245**
	VG-BD-L048	140	77	56	**273**
	VG-BD-L050	0	322	560	**882**
	VG-BD-L069	147	147	56	**350**
	VG-BO-L105	0	126	56	**182**
	VG-BO-L109	0	0	182	**182**
	VG-BO-L113	0	0	161	**161**
	VG-BO-L136	0	0	182	**182**
	VG-BO-L140	0	56	0	**56**
	VG-BO-L142	0	112	56	**168**
	VG-BO-L145	0	0	56	**56**
	VG-LF-L201	1470	1260	1400	**4130**
	VG-LF-L202	1848	567	0	**2415**
	VG-LF-L206	0	0	21	**21**
	VG-LF-L207	1274	742	1323	**3339**
	VG-LF-L210	210	119	126	**455**
	VG-LF-L215	1321	882	1358	**3561**
	**total**	**7418**	**7140**	**8456**	**23014**
caprine	VG-BD-L002	84	21	84	**189**
	VG-BO-L145	0	0	21	**21**
	**total**	**84**	**21**	**105**	**210**
equine	VG-BD-L002	0	42	63	**105**
	VG-BD-L033	0	42	63	**105**
	VG-BO-L109	56	0	0	**56**
	**total**	**56**	**84**	**126**	**266**
ovine	VG-BD-L002	105	0	105	**210**
	VG-BO-L145	0	0	21	**21**
	VG-LF-L207	56	0	0	**56**
	**total**	**161**	**0**	**126**	**287**

**Table 16.** Results of *A. phagocytophilum*, *Borrelia* spp. and *Babesia* spp. in nymphs from field campaigns 1 to 3 and in small mammals from field campaigns 1 to 4. No *Babesia*-positive small mammals were found. n/N, number of positive samples/number of analysed samples; Prev, prevalence in %; 95% CI, in [], 95% Confidence Interval for prevalence.

		**Questing nymphs**			**Small mammals**	
**Site**	**Pathogens**	***A. phagocytophilum***	***Borrelia* spp.**	***Babesia* spp.**	***A. phagocytophilum***	***Borrelia* spp.**
VG	n/N	35/1891	47/1891	51/1891	0/300	6/143
	Prev 95%CI	1.9 [1.2-2.5]	2.5 [1.8-3.2]	2.7 [2.0-3.4]	0.0	4.2 [0.9-7.5]
ZA	n/N	57/2627	78/2627	82/2627	42/608	26/606
	Prev 95%CI	2.2 [1.6-2.7]	3.0 [2.3-3.6]	3.1 [2.5-3.8]	6.9 [4.9-8.9]	4.1 [2.7-5.9]

**Table 17.** Identification of *Borrelia* species in infected nymphs.

**Species**	**VG**	**ZA**
*Borrelia afzelii*	8	16
*Borrelia burgdorferi* sensu stricto	15	13
*Borrelia garinii*	6	20
*Borrelia valaisiana*	10	14
*Borrelia spielmani*	0	1
*Borrelia turdi* or *B. lusitaniae*	0	1
Co-infection	4	6
Non exploitable sequence	4	7
**Total**	**47**	**78**
